# Balancing Work and Schizophrenia: Challenges in Fitness-for-Work Assessment

**DOI:** 10.1192/j.eurpsy.2026.12125

**Published:** 2026-07-31

**Authors:** Y. Tebourbi, G. Bahri, Y. Yaich, G. Amri, M. Mersni, S. Chmengui, D. Brahim, M. Bani, R. Ghachem, N. Ladhari

**Affiliations:** 1 Occupational Medicine and Professional Pathologies Department, Charles Nicolle Hospital, Tunis; 2 Psychiatry Department B, Razi Hospital, Manouba, Tunisia

## Abstract

**Introduction:**

Assessing medical fitness for work in patients with schizophrenia remains challenging due to the disorder’s complex medical, organizational and social implications for both employees and employers.

**Objectives:**

The aim of this study was to describe the medical and occupational characteristics of patients with schizophrenia and analyze the determinants of medical fitness for work decisions.

**Methods:**

A descriptive, retrospective study was conducted on medical records of patients with schizophrenia referred to the occupational health department at Charles Nicolle University hospital in Tunis for fitness-for-work assessment between 2020 and 2024.

**Results:**

Fifteen cases were analyzed. Most participants were male (86.7%) with a median age of 38 years (range: 24-56) and mean professional seniority of 15.2 ±8 years. The most represented sectors of activity were public health (n=3), public administration (n=3), and electronics (n=2). Most patients (86.7%) had no psychiatric history, and 26.7% had been diagnosed with schizophrenia before starting employment. At assessment, 47% were asymptomatic, 26.7% had active psychotic symptoms, and 20% reported sleep disorders Regarding treatment response, 46.7% were well-controlled, 33.3% partially controlled, and 20% poorly controlled. Fitness outcomes were: 6.7% of patients fit for current work, 46.7% fit with workplace adjustments, 33.3% permanently unfit, and 13.3% temporarily unfit. Required adjustments primarily involved removing night shifts (40%), excluding responsibility roles (33.3%), and driving restrictions (6.7%). Determinants of fitness decisions were primarily job characteristics, working hours, and clinical stability.

**Conclusions:**

Fitness-for-work evaluations in patients with schizophrenia present significant challenges requiring careful consideration of multiple medical and occupational factors. These findings, despite the small sample size, highlight the need for clear guidelines to assist occupational physicians.

**Disclosure of Interest:**

None Declared

